# Robustness promotes evolvability of thermotolerance in an RNA virus

**DOI:** 10.1186/1471-2148-8-231

**Published:** 2008-08-11

**Authors:** Robert C McBride, C Brandon Ogbunugafor, Paul E Turner

**Affiliations:** 1 Department of Ecology and Evolutionary Biology, Yale University, New Haven, Connecticut, 06520-8106, USA

## Abstract

**Background:**

The ability for an evolving population to adapt to a novel environment is achieved through a balance of robustness and evolvability. Robustness is the invariance of phenotype in the face of perturbation and evolvability is the capacity to adapt in response to selection. Genetic robustness has been posited, depending on the underlying mechanism, to either decrease the efficacy of selection, or increase the possibility of future adaptation. However, the true effect of genetic robustness on evolvability in biological systems remains uncertain.

**Results:**

Here we demonstrate that genetic robustness increases evolvability of thermotolerance in laboratory populations of the RNA virus φ6. We observed that populations founded by robust clones evolved greater resistance to heat shock, relative to populations founded by brittle (less-robust) clones. Thus, we provide empirical evidence for the idea that robustness can promote evolvability in this environment, and further suggest that evolvability can arise indirectly via selection for robustness, rather than through direct selective action.

**Conclusion:**

Our data imply that greater tolerance of mutational change is associated with virus adaptability in a new niche, a finding generally relevant to evolutionary biology, and informative for elucidating how viruses might evolve to emerge in new habitats and/or overcome novel therapies.

## Background

Evolvability may be defined as the capacity to adapt in response to selection [1-3], or alternatively as the ability to access evolutionary innovations [4,5]. These varied definitions echo the diverse opinions on how evolvability might be influenced by aspects of genetic architecture, especially genetic robustness – phenotypic constancy in the face of mutational change [6]. If robustness affects evolvability, it should impact the ability for organisms to access evolutionary innovations [4,5]. Robustness more easily allows for the accumulation of mutations that are neutral in the current environment; should the habitat change, this robust genetic architecture may then promote access to a relatively greater number of mutations that are beneficial for adaptation [5]. For example, a robust population may be envisioned as residing in a region of a fitness landscape that is relatively flat, owing to the high proportion of resident genotypes in the population that are equal (neutral) in fitness [7]. This creates a large 'neutral network' of genotypes that can efficiently traverse the landscape through random drift, due to their high degree of network connectivity. If environmental change alters the fitness landscape, a robust population may experience an evolvability advantage because newly-arising mutations occur in a wider diversity of genetic backgrounds, creating more-varied epistatic combinations that may prove beneficial for adaptation [8].

Until recently, it was controversial whether biological populations could evolve genetic robustness as posed by theory [9]. However, empirical work confirms that robustness of RNA viruses can be altered through directional selection [10], and that elevated mutation rates in RNA viruses and viroids selects for fitness improvement via increased robustness despite concomitantly reduced replication rate [11,12]. In contrast, the relationship between robustness and evolvability remains elusive; although the literature contains anecdotal accounts of their purported link [5,13], these examples mostly derive from the molecular level of organization [5,14]. Furthermore, these data are inconsistent, with some studies suggesting a positive relationship between robustness and evolvability [14,15] and others implying a negative relationship [16-18]. To date there are no empirical data from biological systems which examine this relationship [5,6]. An ideal approach would be to study the influence of robustness on evolvability, using an empirical system where relatively robust and brittle genotypes have been identified, and which is tractable for studying adaptation under strong selection in a novel habitat.

**Figure 1:**
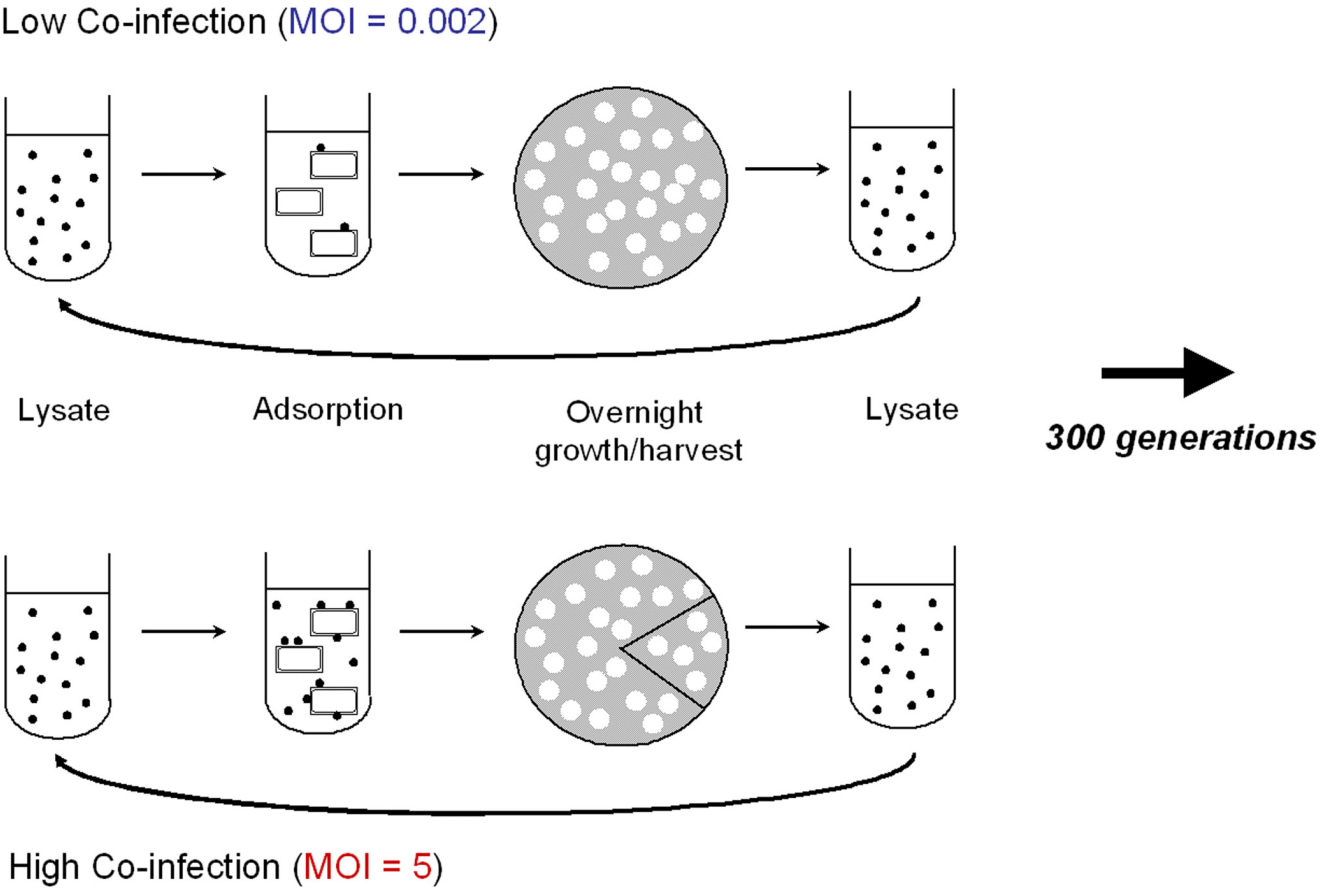
**Summary of the propagation schemes for the low and high co-infection treatments in Turner and Chao (1998).** Phage (●) adsorbed to bacterial cells (□) at a constant multiplicity-of-infection, and this mixture was used to seed a bacterial lawn. During overnight growth, the viral progeny formed visible plaques (○) which were harvested to create a bacteria-free lysate. Plaques in the low co-infection treatment were produced as the result of single infections, whereas those in the high co-infection treatment resulted from co-infection by two to three viruses (on average). To control for differences in population size across treatments, one-fifth as many plaques were harvested in the high co-infection treatment. See text for details.

**Figure 2:**
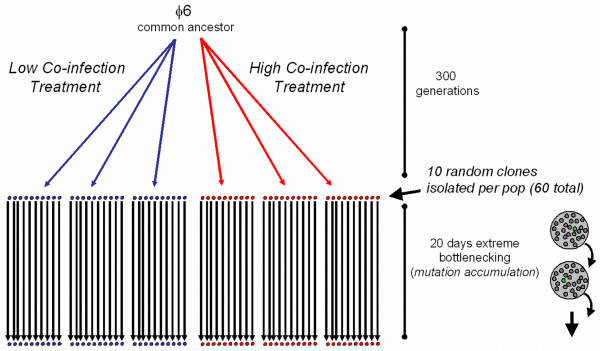
**Design for an evolution experiment where a wild type bacteriophage φ6 ancestor was used to found 3 lineages in a low level of co-infection treatment, and 3 lineages in a high co-infection treatment.** After 60 days (300 generations), 10 clones were isolated at random from each population, and used to found lineages that were subjected to a mutation accumulation experiment. Cartoon at lower right depicts plaque-to-plaque transfers, where propagating the lineage through extreme bottlenecks of a single individual (green plaque) causes drift to overwhelm selection. The mutation-accumulation study was used to reveal whether prior ecological history (low versus high co-infection) affected the evolution of robustness: virus ability to maintain a constant phenotype (fitness) in the face of random mutational change.

To test whether robustness promotes evolvability, we used a collection of genetically robust and brittle strains of the lytic RNA bacteriophage φ6. These strains originally came from an experimental evolution study [10,19], where replicate virus populations were selected on the bacterium *Pseudomonas syringae *pathovar *phaseolicola*, under low versus high levels of virus co-infection (Figure 1). Three of the populations were cultured at a low multiplicity-of-infection (MOI; ratio of infecting viruses to bacterial cells) of 0.002, where ~99.9% of all infected cells should be infected by a single virus [19]. In contrast, the other three lineages were passaged at MOI = 5, where ~97% of infected cells should be infected by two to three viruses (the limit to co-infection in φ6; [20]). Co-infection was controlled by mixing viruses and bacteria at a given MOI, allowing sufficient time for cell adsorption, and then plating a dilution of the mixture onto agar with superabundant cells (Figure 1). During overnight incubation, viruses formed distinct (non-overlapping) plaques, which result from infected cells that lyse and release viral progeny that infect neighboring cells. The passage cycle was repeated by harvesting plaques, removing the bacteria by filtration, and mixing viruses and naïve (non-coevolving) bacteria at the controlled MOI. A total of 60 passage cycles were conducted [10]. Because five generations occur during overnight plaque formation [19], co-infection level was manipulated every fifth generation and the lineages experienced roughly 300 generations of viral evolution (Figure 2) [10]. Thus, the two treatments were equivalent, except that the high co-infection populations more often experienced an environment allowing intracellular virus interactions.

We previously showed that a key intracellular interaction in virus φ6 is genetic complementation [21], where the effects of harmful mutations can be masked by superior protein products provided *in trans*. Thus, the high co-infection lineages benefited from complementation as a 'built-in' robustness mechanism to buffer mutational effects. However, we predicted that this mechanism would lead to weakened selection for the high co-infection viruses to maintain individual-level robustness. The hypothesis was tested in a study where we randomly isolated 10 clones from each of the six previously evolved populations, and used each of these 60 clones to found a single lineage that was subjected to a mutation accumulation experiment [10] (Figure 2). Mutation accumulation was achieved by serially propagating the lineages in a new environment where they experienced extreme population bottlenecks consisting of single-virus passages (i.e. plaque-to-plaque transfers) on *P. phaseolicola *(Figure 2). [Mutation sampling in such experiments is nearly unbiased because genetic drift overwhelms natural selection during the extreme bottlenecks; without selection, all non-lethal mutations can fix with roughly equal probability, regardless of their deleterious, advantageous or neutral effects [22-24]. But because most mutations are deleterious, mutation accumulation experiments tend to cause reduced fitness [22-24].] Serial bottlenecking was imposed for 20 consecutive days, which should cause each lineage to fix roughly 1.3 random mutations of deleterious effect, on average (i.e., genomic mutation rate of virus φ6 is ~0.067 deleterious mutations per generation [24], and 0.067 × 20 bottleneck events ≅ 1.3 fixed mutations [10]). We compared the fitness consequences of mutation accumulation for lineages founded by viruses drawn from the two selection treatments, by measuring the mean magnitude and variance in fitness change that occurred as a result of bottlenecking. Results confirmed the hypothesis that viruses historically evolved under high co-infection were relatively less robust than those evolved under low co-infection, demonstrated by their greater mean magnitude and variance in fitness changes generated by addition of random non-lethal mutations [10]. In this way, we determined that viruses evolved under 300 generations of low co-infection can be defined as genetically robust, whereas those evolved under high co-infection can be considered genetically brittle (less-robust).

Here we tested the relationship between robustness and evolvability by examining a subset of the pre-mutation accumulation strains of virus φ6 (12 low co-infection evolved viruses, 12 high co-infection evolved viruses), and gauging their relative ability to adapt to a novel environment. To do so, we tested whether populations founded by genetically-robust viruses were more evolvable than lineages founded by genetically-brittle viruses, in an environment that imposes strong directional selection. In particular, we explored whether robust virus lineages had the capacity to evolve faster than brittle populations, when viruses must adapt by evolving thermotolerance – resistance to the deleterious consequences of periodic heat shock.

## Results

### Effects of heat shock on virus φ6 survival

**Figure 3:**
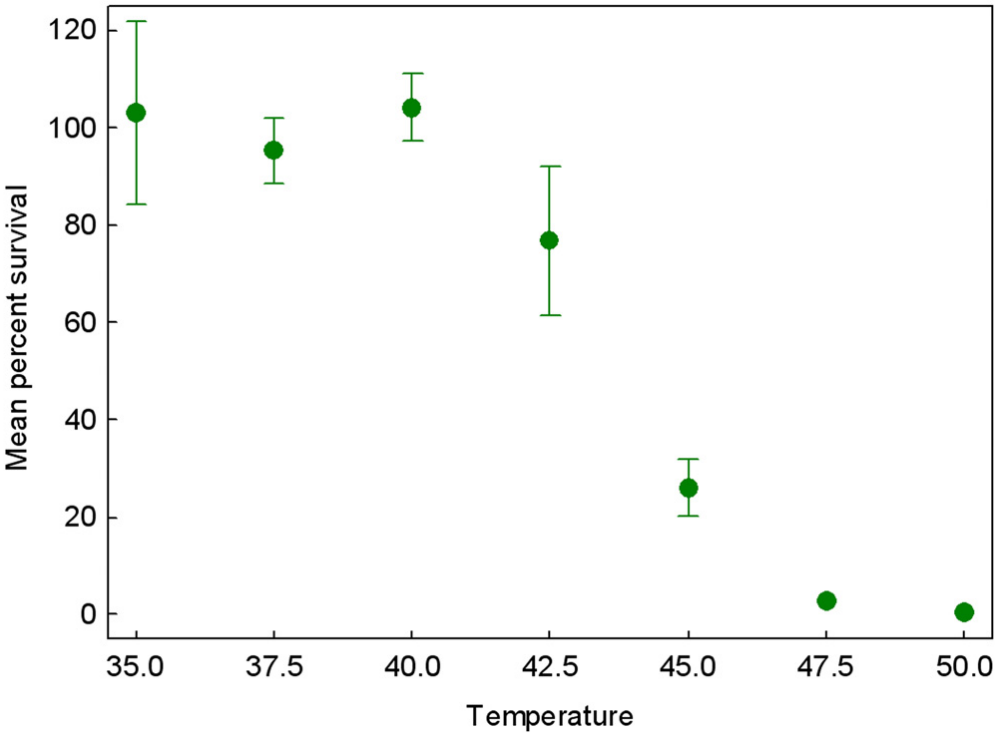
**Percent survival across a thermal gradient for wild type virus φ6 reveals that elevated temperatures are harmful for virus survival.** Each point is the mean of 3 to 5 measures, and error bars are 95% confidence intervals.

To test whether robust viruses are more evolvable than their brittle counterparts, we first identified a novel selective environment which presented a challenge for virus φ6 growth and survival. The standard laboratory temperature for culturing virus φ6 is 25°C. Previous work has shown that the lytic enzyme (protein P5) of virus φ6 is adversely affected by virus exposure to temperatures above 45°C [25]. But the deleterious effects of elevated thermal environments on the virus have not been studied extensively. We developed a survival assay to measure how short term (5 min) thermal incubation affects subsequent ability for a φ6 population to attach to and/or replicate within *P. phaseolicola *host bacteria. Bacteria-free lysates (~10^8 ^virus particles) of the wild type virus were subjected to thermal incubation at seven temperatures ranging between 35°C and 50°C, in repeated (3 <*n *< 5) assays. Results showed that temperatures increasingly higher than 40°C caused a progressively larger fraction of the virus population to become incapable of producing plaques on the host bacteria (Figure 3). These preliminary data indicated that heat shock at an elevated temperature was highly debilitating for the survival and/or fecundity of φ6 virions. Furthermore, the results suggested that propagation of the virus under an elevated temperature should impose strong selection for virus adaptation to resist the deleterious effects of heat shock. We therefore chose 5 min exposure to 45°C as the selective condition to impose heat shock; in this habitat ~80% of wild type φ6 virions become unable to productively infect cells and produce progeny (Figure 3).

### Choice of representative robust and brittle clones

Our prior mutation-accumulation experiment revealed that viruses which were evolved for 300 generations under low and high levels of co-infection could be generally categorized as robust and brittle, respectively [10]. Using the 60-member collection of evolved viral clones that were isolated prior to mutation accumulation, we randomly chose 12 robust clones and 12 brittle clones (i.e., 4 from each low and high co-infection population). To confirm that these clones were representative of the larger collection, we analyzed the pertinent data presented in the earlier study [10]. Each of these clones had been used to found a lineage subjected to 20 consecutive days of mutation accumulation. Subsequently, the change in fitness following random mutational input (Δlog_10 _*W *= log_10 _*W*_post-bottleneck _- log_10 _*W*_pre-bottleneck_) was calculated for each lineage. The data revealed that variance in Δlog_10 _*W *for the 12 lineages founded by high co-infection viruses (σ^2 ^= 0.046) was over twice that of the low co-infection derived lineages (σ^2 ^= 0.020; two-tailed F-test: *P*_0.05,11 _= 0.174). Robustness of one group relative to another can be defined as lesser variance in fitness changes brought on by mutation(s) added to the genome [4,26]. Thus, this result is consistent with the idea that the low co-infection group of viruses can be considered more robust than their high co-infection counterparts [10]. Also, the grand mean Δlog_10 _*W *following mutation accumulation for lineages founded by high co-infection viruses (mean = -0.168) was over three-fold greater than that of the low co-infection derived lineages (mean = -0.052; two tailed t-test: *P*_0.05,23 _= 0.131). This outcome is consistent with theory suggesting that increased mutational robustness should cause mutations to have reduced effects on mean fitness [27]. Although both analyses were consistent with the previous definition of low co-infection and high co-infection viruses as robust and brittle, respectively, we noted that neither analysis showed statistical significance at the 0.05 level. This is perhaps unsurprising because of the reduced statistical power when analyzing differences between two groups of size 12, instead of size 30 as in the earlier study [10]. We concluded that the 24 clones could be used to examine the link between robustness and evolvability, but that the collection would provide a conservative test of the hypothesis.

### Initial survivability of robust and brittle clones under heat shock

**Figure 4:**
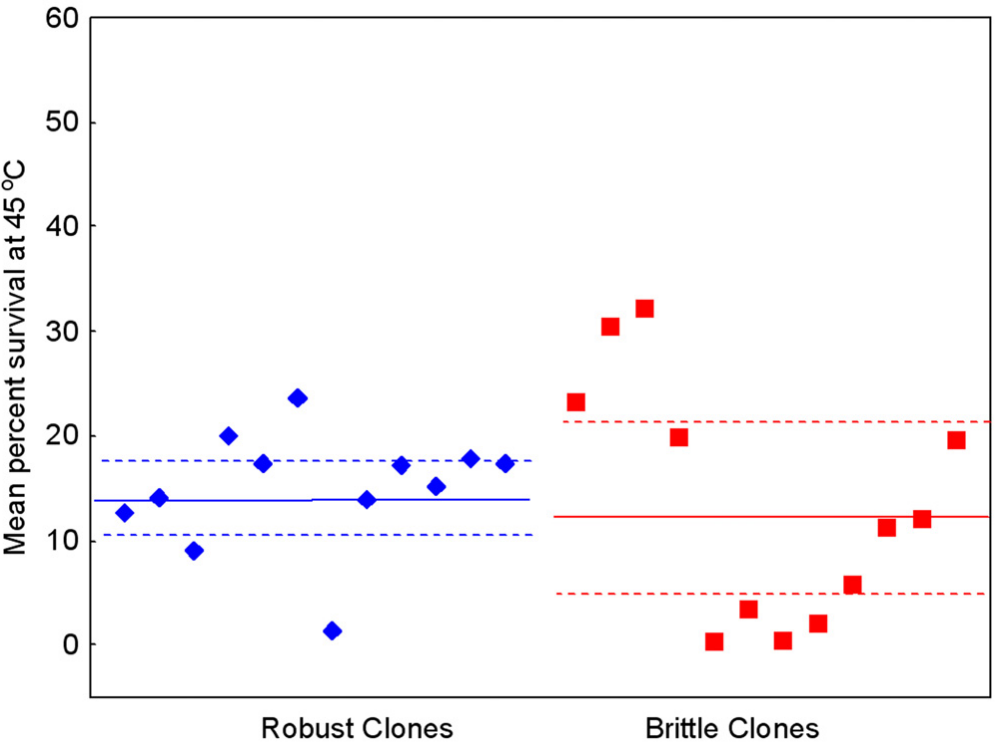
**Initial percent survival after heat shock (45°C) does not differ for 12 robust virus clones (diamonds), and 12 brittle virus clones (squares).** Solid line is the grand mean for the group, and dashed lines are 95% confidence intervals.

We then conducted preliminary assays to confirm that the two groups of strains (12 robust, 12 brittle) were equally poor at surviving the selective heat-shock environment. To do so, we measured the mean of percent survival (%*S*) following 5 min exposure to 45°C for all 24 strains, using repeated (*n *= 6) survival assays. Results (Figure 4) showed that the grand mean %*S *for the 12 low co-infection evolved (robust) strains was 14.899 ± 1.641 s.e.m, and that for the 12 high co-infection evolved (brittle) clones was 13.467 ± 3.312 s.e.m. The average of these data across all 24 strains was 14.182 ± 2.163 s.e.m., which did not differ according to robust versus brittle categorization (two tailed t-test: *P*_0.05,23 _= 0.702). We concluded that the two groups of founding viruses were equivalent in average survival under heat shock at 45°C, regardless of their prior history of selection at low versus high co-infection.

### Equivalent fitness of robust and brittle clones at differing co-infection levels

**Figure 5:**
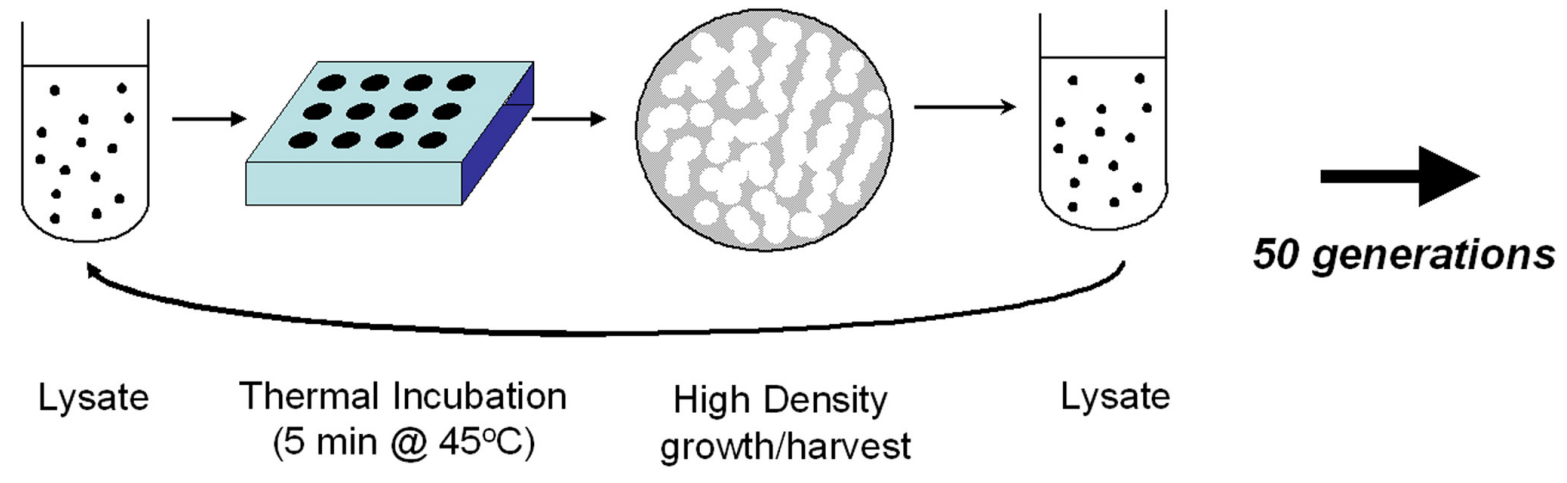
**Design for an evolution experiment where bacteriophage φ6 lineages were selected to withstand damaging effects of heat shock.** A virus lysate was exposed to 45°C incubation for 5 minutes, and a dilution of the surviving progeny was plated on a lawn of *P. phaseolicola *bacteria. Overnight plaque formation at the standard temperature of 25°C corresponded to 5 generations of virus evolution. The plaques were then harvested to remove bacteria, and the process was repeated. Duration of the experiment was 10 days, equivalent to 50 virus generations.

We next conducted two preliminary experiments to determine whether the groups of viruses differed in fitness (relative growth on *P. phaseolicola*) at low and high co-infection. Whereas the design of the original 300-generation experiment allowed pre-adsorption to cells to manipulate co-infection level (Figure 1), the thermal selection regime omitted this step so that individual viruses generally infect cells on their own (Figure 5). We noted that this elimination of co-infection might cause the thermal selection to more closely mimic the previous low co-infection habitat. In particular, if the high co-infection strains were relatively poor at performing at low MOI, it would suggest that they may be additionally challenged by simultaneously adapting to both heat shock *and *low co-infection in the planned evolvability experiment. To determine whether the two groups of founding viruses differed in performance at low co-infection, we analyzed previous data on the mean log_10 _*W *of each of the 24 strains relative to a common competitor, obtained through repeated (*n *= 3) fitness assays. Consistent with earlier data for the entire 60-member collection [10], results for these 24 clones showed that mean log_10 _*W *at low MOI did not significantly differ according to prior adaptive history (low versus high co-infection) (two tailed t-test: *P*_0.05,23 _= 0.856). We concluded that the intended thermal selection regime involving propagation in absence of co-infection would not bias in favor of viruses previously adapted to low co-infection conditions.

For completeness, we also determined whether the two groups of founding viruses differed in performance at high co-infection, by estimating the mean log_10 _*W *of each of the 24 strains relative to a common competitor, using repeated (*n *= 3) fitness assays. Results showed that mean fitness did not significantly differ according to prior adaptive history (low versus high co-infection) (two tailed t-test: *P*_0.05,23 _= 0.135). We concluded that the two groups of viruses were equivalent in fitness under low and high levels of co-infection (see further discussion below).

### Robust viruses are more evolvable under thermal selection

To test the formal hypothesis that evolvability correlates with robustness we used the 12 robust and 12 brittle clones as founders of independent lineages in an experimental evolution study. The 24 lineages were subjected to 50 generations (10 days) of viral evolution, involving daily exposure to 5 min incubation at 45°C, followed by five generations of growth on *P. phaseolicola *at 25°C. Thus, heat shock was imposed only every fifth generation of virus evolution. At the end of the experiment, we used replicated (n = 6) survival assays to measure mean %*S *at 45°C for each of the founding clones, and for each of their corresponding derived endpoint populations. Measurements of %*S *for a founding genotype and its evolved descendant population were conducted in parallel. The difference between the two mean values was then used to estimate Δ%*S*, the change in average percent survival at 45°C following 50 generations of viral evolution with periodic heat shock.

**Figure 6:**
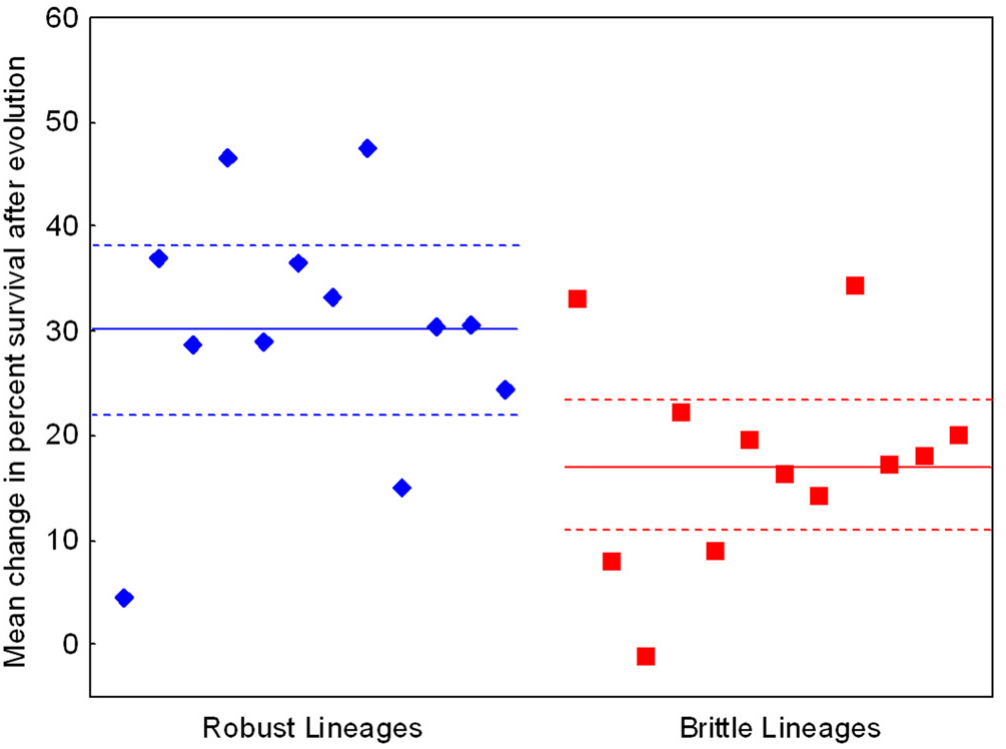
**Mean change in percent survival after heat shock (45°C) is greater for virus lineages founded by 12 robust strains (diamonds), relative to that for lineages initiated by 12 brittle strains (squares).** All populations were subjected to 10 days (50 generations) of selection in a high temperature environment. Solid line is the grand mean for the group, and dashed lines are 95% confidence intervals.

Results (Figure 6) showed that the mean Δ%*S *for lineages founded by the robust strains was a significantly larger value than that for the populations initiated by brittle strains (Wilcoxon/Kruskal Wallis, *n *= 24, *P *= 0.016). Although the founding clones showed no difference in heat-shock survival prior to selection (Figure 4), we noted that subtle differences in initial survival might be an important factor in determining the extent of thermal adaptation shown in Figure 6. We thus conducted a separate analysis to determine whether final Δ%*S *achieved by an experimental lineage correlated with the initial %*S *of its corresponding ancestral clone. The analysis confirmed that these variables were not significantly correlated (correlation: *r *= 0.036, *P *= 0.868). Overall, these data provided strong support for the hypothesis that genetic robustness promotes evolvability of virus φ6 in a novel habitat where heat shock imposes directional selection.

Aside from the imposition of periodic heat-shock, the selection experiment was designed to be highly similar to the general culture conditions that the founding clones previously experienced during their 300 generations of evolution under low versus high co-infection (cf. Figures 1 and 5). To confirm that the periodic heat shock was indeed providing the expected strong directional selection, we conducted an additional selection experiment using a subset of the founding clones that did not differ in initial %S. In this control experiment the lineages experienced 10 passage days (50 generations of virus evolution), but using 5 min incubation at the typical 25°C culture temperature every fifth generation. At the end of the experiment we measured Δ%*S *at 45°C with three-fold replication, for the founding strains and their descendant populations in parallel. Results showed that the two groups of evolved lineages in this control experiment did not statistically differ in Δ%*S *at 45°C (two tailed t-test: *P*_0.05,11 _= 0.687). We therefore concluded that change in resistance to heat shock was indeed an adaptive trait, and not simply a consequential response to the general conditions manipulated in our experiment to address the formal hypothesis.

## Discussion

Our results showed that variants of RNA virus φ6 that were previously determined to be advantaged in their relative genetic robustness [10], were also advantaged in terms of their relative evolvability: greater capacity to undergo adaptive change in a novel environment. These data provide empirical evidence that increased robustness can promote evolvability in a biological system, at least for an RNA virus challenged by growth under the heat-shock conditions we imposed.

### Differential mutability is an unlikely mechanism driving evolvability

We previously suggested that lower replication fidelity should be more easily tolerated by biological systems which are relatively robust, or phenotypically constant, in the face of mutational change [10]. In this way, evolution of higher mutation rate may be expected to coincide with evolution of genetic robustness. In turn, this elevated mutation rate could provide an underlying genetic mechanism to explain why robustness promotes evolvability. That is, the increased genetic variation which is concomitant with high mutation rate may prove useful under environmental change, assuming that the greater variation affords access to more mutations of beneficial effect. We note that this suggested mutational mechanism linking robustness and evolvability is fundamentally different than the well-known advantage of mutator genotypes for hastening the adaptive process [28,29]. Mutator mutants may be found at higher than expected frequencies in laboratory and natural populations of bacteria, because mutators profit from their increased production of beneficial mutations by genetic hitch-hiking when populations adapt to novel conditions [30]. Once a population becomes dominated by mutators its fate is unclear, however. The long-term fitness of mutator populations may be low for many reasons, such as their tendency to experience mutations that eliminate functions unneeded in the current environment but essential in future habitats, and their risk of mutational extinction when population size is small [30]. Thus, mutator mutants (or mutator alleles) may only provide a transient competitive advantage. In contrast, we suggest that the coupling of increased mutation rate with robustness should be less of a liability; e.g., a high fitness load of deleterious mutations is unexpected in robust systems because they are defined as relatively buffered against mutation effects [4]. In this way, elevated mutation rate paired with robustness may provide a better strategy than mutators, for high mutation rate to promote evolvability.

Our earlier work hinted that elevated mutation rate may be a characteristic of the virus φ6 strains which we defined as relatively robust [10]. In particular, we examined a set of 3 robust and 3 brittle clones (1 drawn from each population at generation 300), and compared their ability to generate spontaneous mutants that were capable of growing on two novel species of host bacteria (*P. syringae *pathovar *atrofaciens*, *P. syringae *pathovar *tomato*). We observed that the robust viruses tended to generate much higher frequencies of mutants, which suggested that robustness coincided with a higher mutation rate [10]. However, in that same study we cautioned that the mutation frequency data may not be generally representative of changes occurring at other loci; although many different mutations underlie the expanded host-range phenotype, these changes generally map to the P3 gene on the medium RNA segment of virus φ6 [31-33]. Furthermore, our data also showed that the higher mutation frequency of the robust viruses became greatly diminished (but still statistically differed), when viruses first mutated to infect *P. atrofaciens *and were then forced to obtain an additional mutation that allowed infection of *P. tomato *[10]. The latter results strongly suggested that a two-step mutation process governed the host-range phenotype and that the robust viruses happened to possess the precursor allele change, whereas the brittle viruses did not. Therefore, the data may be more indicative of interesting differences in genetic architecture of gene P3 among robust and brittle strains, but these differences may confound comparisons of their relative mutation rates.

**Figure 7:**
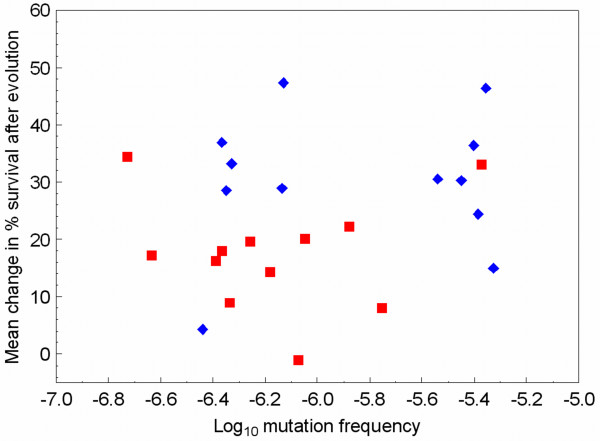
**Changes in percent survival of lineages subjected to heat-shock selection do not correlate significantly with their founding clone's log_10 _mutation frequency for BHT resistance, a proxy for mutation rate.** Data are for the 12 lineages and founding clones defined as robust (diamonds), and as brittle (squares).

We identified a method for comparing mutant frequencies among robust and brittle strains of virus φ6 which should be less confounded by any of their underlying differences in genetic architecture in gene P3. The approach involved examining the frequency of mutants that are resistant to the chemical butylated hydroxytoluene (BHT). BHT is shown to be lethal to virus φ6 genotypes because it cleaves protein P3 from the virion, preventing attachment and subsequent entry of the virus into the *P. phaseolicola *host cell [34]. But one or more spontaneous mutations in gene P6 can provide resistance to BHT, because protein P6 anchors protein P3 and certain mutations in P6 prevent the cleavage process [34]. Using lysates of the 24 clones that founded our selection experiments, we measured the ability for a clone to generate mutants resistant to BHT (see Methods). We observed no significant difference in the frequency of BHT resistant mutants according to categorization of the clones as robust versus brittle (Wilcoxon/Kruskal Wallis, *n *= 24, *P *= 0.355). Because variability in mutant frequency was seen among strains in these assays, we surmised that subtle differences in BHT-mutant frequency (as a proxy for mutation rate) might be associated with the observed differences in evolvability. Thus, we also examined the relationship between BHT-mutant frequency and mean adaptive change in our heat-shock experiment (Δ%*S*) for the set of 24 clones. This analysis (Figure 7) showed no overall significant correlation between the two variables (correlation: *r *= 0.244, *P *= 0.251). In addition, correlation analyses conducted independently for the two groups were not significant either (12 robust clones: *r *= 0.112, *P *= 0.730; 12 brittle clones: *r *= 0.013, *P *= 0.967). These results strongly suggested that mutation rate differences among strains in our study did not influence their observed evolvability. Furthermore, these data for BHT mutant frequencies do not provide support for elevated mutation rate as the underlying mechanism governing the observed link between robustness and evolvability in virus φ6. However, although we cautioned against using host-range assays to discern mutation rate differences among robust and brittle clones [10], we note that these results combined with the current BHT-resistance assays provide mixed evidence for the hypothesis on differential mutability. We therefore do not unequivocally reject the idea that higher mutation rates contribute to the evolvability advantage of robust lineages under heat shock.

A somewhat related idea is that adaptation to the 45°C heat shock might require multiple mutational substitutions, and the robust clones evolved relatively faster because they happened to contain one or more of the needed mutations. We find this explanation unlikely, however. We observed that the initial survival of robust and brittle clones did not statistically differ at 45°C, using replicated survival assays which exposed lysates containing 10^8 ^particles of a test clone to the challenge temperature (see Methods). These assayed lysates should be easily large enough to harbor rare spontaneous mutants; although the mutation rate and mutation spectrum for 45°C heat-shock resistance are unknown in φ6, we can expect between 10^-4 ^and 10^-6 ^particles should contain a point mutation for resistance to a selective challenge such as growth on a novel host [21,35]. Thus, if the genetic architectures of robust clones happened to place them much closer to the needed optimum (e.g. only one mutation away), rare mutants with high percent survival should occur more often in the assays involving robust clones, producing occasional "jackpots" in survivorship of these clones and a detectable difference in mean survivability between robust and brittle strains. We did not observe such greater variance among assay replicates at 45°C for the robust clones (data not shown); therefore, the available data do not support the idea that robust strains are relatively fewer mutations away from the optimum.

### Differential protein stability as a potential mechanism for robustness and evolvability

We have yet to determine the exact molecular mechanism responsible for differences in genetic robustness among φ6 virus genotypes. However, data from other systems point to differences in protein stability as the responsible mechanism. In particular, it has been shown that proteins can be identical in shape, despite underlying sequence differences that impact protein sensitivity to the effects of mutations (i.e. robustness) [15]. These results suggest that the less-sensitive (relatively robust) proteins are more likely to maintain their function in a new environment where innovation is needed [15,36]. This mechanism may similarly account for differences in robustness among strains of virus φ6, and could explain why robustness is linked with evolvability. That is, the robust viruses may feature proteins that tend to be capable of undergoing mutations while maintaining their proper folding. In the heat-shock environment we provided as a selective challenge, evolution of greater protein thermostability would be beneficial. We infer that one or more proteins of the robust viruses were better adept at maintaining their proper fold, while allowing the input of spontaneous mutation(s) that led to exploration of novel thermostable genotypes. This combination would thus explain the increased ability for the robust viruses to adapt to the heat shock. In contrast, the brittle viruses were perhaps constrained in their ability to adapt because their proteins encountered mutations that increased thermostability, while compromising the ability to efficiently produce viral progeny.

We showed that the 12 robust and 12 brittle viruses used as founding genotypes in the current study were equally poor at surviving in the 45°C selective habitat (Figure 4). But empirical support for the argued protein stability mechanism would be increased survivability of robust strains at less-extreme temperatures, such as those ranging between 40°C and 45°C. Our preliminary data for a subset of the strains support this idea because the robust genotypes showed relatively greater survival at temperatures such as 42°C (Ogbunugafor, McBride and Turner, unpublished data); however, future work is needed to confirm the presumed thermostability advantage of robust strains. We note that percent survival at 42°C was variable among the subsets of 6 robust and 6 brittle clones examined in these preliminary assays, and that 42°C survival did not correlate with their observed evolvability (Δ%*S *at 45°C) in our selection experiment (robust strains: *r *= 0.222, *P *= 0.672; brittle strains: *r *= 0.387, *P *= 0.448). Therefore, our data suggest that robust strains of φ6 are generally better at surviving moderate temperatures such as 42°C, but individual strain toleration of this temperature does not predict the adaptive success of its descendent lineage under 45°C selection. This analysis suggests that virus performance at moderate temperatures and evolvability under extreme temperature is not a trivial relationship, such as that resulting from a pre-existing mutation in a founding clone which places its descendent lineage closer to a selective optimum that is only two mutational steps away.

### Choice of additional environments to study the robustness/evolvability link

If we are correct in inferring that robust genotypes of virus φ6 tend to contain proteins which are relatively thermostable, one might predict that the positive relationship between robustness and evolvability is specific to elevated-temperature environments, such as the 45°C heat-shock selection imposed in the current study. But this is unknown. Thus, it would be interesting and useful to examine the link between robustness and evolvability of virus φ6 in novel environments aside from 45°C heat shock, but this effort extends beyond the scope of the current study. The virology literature describes many examples of how virus growth/survival can be negatively affected by environmental perturbations [37-39]. For instance, viruses can be damaged due to abiotic challenges such as increased acidity or UV irradiation, and virus growth can be restricted by biotic challenges such as infection of novel host species or cell types [37-41]. Thus, there exist varied and abundant choices for examining the relationship between robustness and evolvability in bacteriophage φ6 and in other virus systems.

But our study suggests that caution is warranted when choosing an environment that may be appropriate for studying the relationship between robustness and evolvability. In particular, our data regarding mutation frequencies on novel host bacteria suggest that differences in underlying genetic architectures exist among robust and brittle strains of virus φ6, constraining the ability for brittle viruses to undergo spontaneous mutations allowing use of novel *Pseudomonas *hosts [10]. Thus, in our particular virus system the choice of a novel host habitat for examining the relationship between robustness and evolvability may be particularly problematic. We infer that differential changes in the genetic architecture of P3 among low and high co-infection evolved viruses may simply have been a consequence of evolution in these two habitats. For example, the robust strains which evolved under low MOI perhaps needed to maintain stability of the P3 protein as part of their inherent robustness, whereas this gene could have changed in the brittle strains because the high MOI environment itself (i.e., genetic complementation) provided the needed robustness. Whatever the reason for this apparent difference in genetic architecture, it indicates that evolution of novel host-use may be a troublesome context for examining the relationship between robustness and evolvability in virus φ6.

### Resolving fitness trade-offs and the evolution of robustness

We inferred that individual-level robustness de-volved in the high co-infection selected viruses due to operation of genetic complementation which provided a "built-in" environmental mechanism for robustness [10]. But we also asserted that the occurrence of complementation caused selection to favor the evolution of "cheater" genotypes of virus φ6 in the high co-infection lineages [19]. The evidence was that viruses evolved under high co-infection (but not low co-infection) improved in fitness relative to the ancestor in their selective environment, at the expense of fitness improvement in the unselected environment [19]. This performance trade-off was especially evident after 200 generations of selection, when cheater viruses seemed to dominate the high co-infection lineages; these cheaters gain a fitness advantage when co-infecting a cell with non-cheater genotypes, but perform relatively poorly when infecting a cell on their own [42-44]. However, the current study confirms our earlier observation that by generation 300 the low and high co-infection evolved clones are equivalent in fitness across the two environments [10]. We suggested that this result could be explained by dissimilarities between average fitness of a microbial population, compared with the fitness of clones drawn from the parent population [10].

However, here we offer a more plausible (and simpler) explanation for the transient nature of the trade-off suffered by high co-infection evolved viruses. The possibility exists that by generation 300 the high co-infection populations are no longer dominated by cheater viruses which present the trade-off; we discussed this possible outcome in a separate study [43]. If the environment allows co-infection, virus φ6 cheaters can displace the wild type ancestor, consistent with cheating behavior as an evolutionary stable strategy (ESS) in game theory contexts pitting evolved cheaters against the ancestral cooperator [42]. But evolved cheaters cannot prevent invasion by evolved cooperators, indicating a mixed ESS [43]. The ability for cheaters to be tolerated as a minority subpopulation is often seen in biological systems [45]. Thus, the transition of cheater viruses from a majority genotype at generation 200 to a minority subpopulation at generation 300 would explain why random clones drawn from the endpoint high co-infection lineages no longer presented the expected fitness trade-off across environments. Our preliminary results support this idea (Duffy, Dennehy and Turner, unpublished data). Whole genome sequencing was used to identify the molecular substitutions which distinguish the generation-200 cheater viruses from the wild type ancestor and from the similarly evolved cooperator viruses. By generation 300, none of these characteristic substitutions are observed as majority alleles in population-level consensus sequencing of the parent populations (Duffy, Dennehy and Turner, unpublished data).

### Evidence for increased genetic diversity within robust populations

Genetic analyses should also prove useful for confirming the presumed role of latent genetic variation in our observed link between robustness and evolvability. Regardless of the molecular mechanism (e.g. protein stability) responsible for robustness in virus φ6, it is expected that a robust population should harbor more genetic diversity than its brittle counterpart. This assumption follows directly from the definition of robust genomes; i.e. they should be more easily capable of amassing neutral mutations because their robustness causes random mutations to be neutral in the current environment. All else being equal, we therefore might expect a larger number of molecular substitutions per genome in the generation-300 strains drawn from the low co-infection lineages, and a greater number of haplotypes per population in these treatment populations. Of course, the problem is that all else is not necessarily equal. Immediately above we discussed how cheaters might have been transiently successful in the high co-infection lineages, which suggests that virus-virus coevolution in these populations might have caused greater dynamic turnover of alleles and, hence, more extensive molecular evolution.

Nevertheless, we acquired preliminary sequence data to examine the predicted larger number of substitutions/strain, and larger total number of haplotypes present (i.e. greater genetic diversity) in the generation-300 lineages evolved under low co-infection, which we defined as relatively robust. As part of a separate study (Duffy, Dennehy and Turner, unpublished data), for all 24 strains we sequenced a 665 bp region on the large segment which is entirely protein-coding for the RNA-dependent RNA polymerase P2, a region that is thought to be highly conserved [46,47]. We also sequenced a 658 bp region on the small segment containing the C-terminal end of the lytic protein P5; the majority of this region (438 bp, or 66.6%) is not protein-coding [48] and may be under weaker selection because it seems to be the least translated portion of the virus φ6 genome [49,50]. Together the two regions represent ~10% of the total φ6 genome.

As expected, very few changes were observed in the sequenced portion of the highly-conserved polymerase gene P2. Two synonymous substitutions were found in this protein-coding region of the large RNA segment: mutation N104N shared by clones L2.3 and L2.4, and mutation E255E shared by clones H1.1 and H1.7. Few changes were also observed in the coding region of the sequenced portion of gene P5 on the small segment. Here we observed two synonymous substitutions (F169F shared by clones L1.9 and L3.4; P197P shared by clones L2.8, H3.1, H3.5, H3.6 and H3.9) and one non-synonymous change (A208E in clones H1.3 and H1.6). The sequenced non-coding region of gene P5 showed seven total mutations, all of which occurred in five clones drawn from populations in the low co-infection treatment: t2209c in L3.2, g2231a in L1.9, a2272g in L1.2, c2281t in L2.3 and L2.4, t2387c in L2.3 and L2.4. These five non-coding mutations explain the difference in the number of mutations observed in clones drawn from the two treatments (eight unique mutations in the low multiplicity evolved clones, three in the high multiplicity strains). The data show that the low co-infection (robust) strains had an average of 1.08 substitutions/strain (13 mutations across 12 clones), compared to 0.58 substitutions/strain (7 mutations across 12 clones) for the high co-infection (brittle) strains. The sequence data also allowed us to estimate the inferred number of haplotypes per evolved population: L1, 3; L2, 3; L3, 3; H1, 2; H2, 1; H3, 1. The difference between robust and brittle populations in the inferred number of haplotypes was found to be statistically significant (two-tailed t-test: *P*_0.05,4 _= 0.007). We concluded that the φ6 populations evolved under low multiplicity showed greater genetic variability than the high MOI populations, on average, based on sequence data representing ~10% of the viral genome.

Although these results are highly preliminary, they are consistent with the expected influence of robustness on genetic variation within populations. Finally, we note that this apparently greater genetic variance among the 12 robust clones did not translate to greater variance in survivability under 45°C heat shock; rather, the less-diverse brittle clones were observed to be more variable in initial thermotolerance (Figure 4). This interesting outcome is entirely consistent with the greater constancy of phenotype expected for robust genotypes relative to brittle ones, despite the greater genetic variance predicted under robustness.

## Conclusion

Our empirical data are highly valuable for advancing general understanding of evolutionary biology, because we demonstrated that robustness and evolvability can be positively correlated in a biological system. Also, we cautiously suggest that our results may be broadly relevant to the evolution of RNA viruses in general. Attention is often focused on the medical importance of RNA viruses, and the ease with which these pathogens seem to emerge in humans. New or improved antiviral drugs are becoming increasingly crucial for controlling RNA viruses, because vaccines are often unavailable or ineffective in treating infections and disease. It is believed that some of these drugs are effective because they elevate RNA virus mutation rate within the host individual, perhaps owing to mutational meltdown that causes the virus population to go extinct [51]. Although such therapies can effectively decrease viral fitness, they also may strongly select for evolution of mechanisms allowing virus resistance [52]. One possible mode of resistance is reduced sensitivity to the deleterious effects of elevated mutation rates via increased robustness [11], as suggested in the evolution of HIV-1 populations [53]. However, the likelihood of such evolved mechanisms remains largely unexplored. Our study warns that increased resistance to mutational therapies may simultaneously select for RNA viruses that have a greater potential to adapt to future therapies, suggesting caution should be heeded when considering the usefulness of such interventions.

By suggesting that robustness may positively relate to evolvability, our study sheds light on a fundamental tension that exists in explaining how organisms persist in the face of environmental change. The ability to withstand change, while simultaneously adapting to future unknown perturbations are tasks whose simultaneous achievement have heretofore seemed incongruous. Our study hints that these seemingly incompatible tasks can be achieved in a biological system, and demonstrate how evolution itself has the potential to evolve.

## Methods

### Strains and Culture Conditions

Virus φ6 is a lytic bacteriophage which contains a ~13 kb double-stranded RNA genome divided into three segments per particle [54]. The current study used 24 clones of virus φ6 that were derived from six experimental lineages (4 clones per lineage) in a previous study examining the effects of low versus high co-infection on virus evolution (Figures 1, 2) [10,19,42,43]. The 12 low co-infection-evolved clones were previously described as robust (L1.10, L1.2, L1.7, L1.9, L2.3, L2.4, L2.6, L2.8, L3.2, L3.4, L3.7, L3.8), and the 12 high co-infection-evolved clones as brittle (H1.1, H1.3, H1.6, H1.7, H2.10, H2.2, H2.4, H2.5, H3.1, H3.5, H3.6, H3.9), based on data amassed for experimental lineages founded by these clones in a separate mutation-accumulation study (Figure 2) [10]. *Pseudomonas syringae *pv.*phaseolicola *(American Type Culture collection #21781) was the host bacterium used in all experiments, and culture conditions are previously described [10,55]. Bacterial stocks were stored in 4:6 glycerol/LC (v/v) at -80°C. Viruses were grown on lawns made from overnight bacterial cultures. Agar concentrations in plates were 1.5% and 0.7% for bottom and top LC agar, respectively. Plates contained 3 mL of top agar and a 200 μL bacterial lawn. Virus lysates were prepared by growing viruses on a *P. phaseolicola *lawn for 24 hr; plaques were then collected and filtered (0.22 μm filter, Durapore, Millipore) to remove bacteria. Virus lysates were stored at -20°C in 4:6 glycerol/LC (v/v).

### Survival Assays

Temperature survival was assayed by placing 120 μl of a virus lysate (~10^8 ^particles) in a PCR tube, followed by immediate sampling onto a host lawn to confirm the initial virus titer (*N*_i_). The lysate was then placed in a preheated Eppendorf thermocycler for 5 min incubation, followed by sampling onto a host lawn to measure final titer (*N*_f_). Percent survival (%*S*) equaled (*N*_f_/*N*_i_) * 100. Thus, survival at a test temperature was gauged through plaque-forming units (pfu), the phage particles that were viable for growth at 25°C.

### Heat-Shock Selection

Virus lineage adaptation to heat shock occurred through experimental evolution involving periodic exposure to high temperature (Figure 5). A lineage experienced the survival assay at 45°C, followed by sampling to create a dilution series on host lawns. After overnight incubation at 25°C, the dilution yielding ~10^3 ^pfu was harvested to obtain a new lysate. The survival assay and plating were then repeated using naïve (non-coevolved) bacteria. This propagation scheme was repeated for 10 consecutive days. Because the overnight plaque growth corresponded to 5 viral generations [10], the experiment lasted 50 generations, where heat shock occurred every fifth generation. A total of 24 virus lineages (12 robust, 12 brittle) experienced this evolution with periodic 45°C heat shock. Also, we conducted an otherwise identical control experiment, where a subset of the clones (6 robust, 6 brittle) were allowed to evolve using a periodic temperature challenge of the standard 25°C environment.

### Fitness assay

Fitness assays consisted of paired-growth experiments [22], which compared 24 hr growth on *P. phaseolicola *of a test genotype (or population) relative to a common competitor of φ6 bearing a genetic marker. In some assays the marked competitor was a host-range mutant containing a substitution in gene P3 of the medium RNA segment, allowing infection of *P. pseudoalcaligenes *bacteria. In other assays the competitor was an engineered mutant bearing the alpha subunit of the *Escherichia coli *beta-galactosidase (β-gal) gene on the large RNA segment [21]; fitness comparisons among viruses were only conducted when the strains were competed against an identical common competitor. The test strain and common competitor were mixed at a 1:1 volumetric ratio, and then a dilution of this mixture containing ~400 viruses was plated on a *P. phaseolicola *lawn. Pre-adsorption to cells before plating was only allowed if the fitness assay examined impact of co-infection on relative fitness. After 24 hr incubation, the ~400 plaques were harvested and filtered to obtain a cell free lysate. When the common competitor was a host-range mutant, the ratios of competing genotypes in the starting mixture (*R*_0_) and in the harvested lysate (*R*_1_) were obtained by plating on mixed lawns of *P. phaseolicola *and *P. pseudoalcaligenes *(200:1 mixture), where ordinary and host-range genotypes form turbid and clear plaques, respectively. When the common competitor contained the β-gal gene insertion, ratios were tracked by plating on lawns of *P. phaseolicola *bacteria containing the beta subunit of the β-gal gene, allowing the marked competitor to produce blue plaques on agar containing X-gal (0.4% w/v). Fitness (*W*) was defined as the relative change in ratio of ordinary to marked virus, or *W = R*_1_*/R*_0_.

### Mutant frequency estimates

We measured the frequency of spontaneous BHT-resistant mutants that occurred within virus populations grown in the absence of the chemical. A high-titer lysate (typically ~10^10 ^viruses per mL) of a virus genotype was grown and titered on *P. phaseolicola*, and a sample of the lysate was then placed in 0.12 mM BHT for 5 min. Mutant frequency was calculated as the number of plaque-forming mutants per viruses in the inoculum following BHT exposure.

### Sequencing

Genomic extraction was performed using QIAamp Viral RNA minikits (Qiagen). The dsRNA genome was reverse transcribed using random hexamer primers (Invitrogen, Carlsbad, CA) and resultant cDNA was used as PCR template (primers available on request). PCR products were purified using the QIAquick PCR Purification kit (Qiagen) or ExoSAP-It (US Biological, Swampscott, MA). Sequencing was performed using the BigDye Terminator reaction v3.1 on an ABI 3100. Sequence reads were curated by eye using Sequencher software (ver. 4.2.2, Gene Codes Corporation, Ann Arbor, MI) and compared using ClustalX http://bips.u-strasbg.fr/fr/Documentation/ClustalX/ and MacClade 4.06 (Sinauer Associates Inc., Sutherland, MA).

## Authors' contributions

RCMcB, CBO and PET were involved in the conception, design and writing of this study. RCMcB conducted the experiments and analyses. All authors read and approved of the final manuscript.

## References

[B1] Wagner GP Altenberg L Perspective: Complex adaptations and the evolution of evolvability Evolution 1996 50 3 967 976 10.2307/2410639 28565291

[B2] Kirschner M Gerhart J Evolvability Proceedings of the National Academy of Sciences of the United States of America 1998 95 15 8420 8427 10.1073/pnas.95.15.8420 9671692 PMC33871

[B3] Burch CL Chao L Evolvability of an RNA virus is determined by its mutational neighbourhood Nature 2000 406 6796 625 628 10.1038/35020564 10949302

[B4] de Visser JAGM Hermisson J Wagner GP Meyers LA Bagheri HC Blanchard JL Chao L Cheverud JM Elena SF Fontana W Gibson G Hansen TF Krakauer D Lewontin RC Ofria C Rice SH von Dassow G Wagner A Whitlock MC Perspective: Evolution and detection of genetic robustness Evolution 2003 57 9 1959 1972 14575319 10.1111/j.0014-3820.2003.tb00377.x

[B5] Wagner A Robustness and evolvability in living systems Princeton studies in complexity 2005 Princeton, N.J. , Princeton University Press xii, 367 p.

[B6] Lenski RE Barrick JE Ofria C Balancing robustness and evolvability PLoS Biology 2006 4 12 2190 2192 10.1371/journal.pbio.0040428 PMC1750925 17238277

[B7] Wilke CO Selection for fitness versus selection for robustness in RNA secondary structure folding Evolution 2001 55 12 2412 2420 11831657 10.1111/j.0014-3820.2001.tb00756.x

[B8] Huynen MA Stadler PF Fontana W Smoothness within ruggedness: The role of neutrality in adaptation Proceedings of the National Academy of Sciences of the United States of America 1996 93 1 397 401 10.1073/pnas.93.1.397 8552647 PMC40245

[B9] Hermisson J Wagner GP The population genetic theory of hidden variation and genetic robustness Genetics 2004 168 4 2271 2284 10.1534/genetics.104.029173 15611191 PMC1448756

[B10] Montville R Froissart R Remold SK Tenaillon O Turner PE Evolution of mutational robustness in an RNA virus PLoS Biology 2005 3 11 1939 1945 10.1371/journal.pbio.0030381 PMC1275523 16248678

[B11] Codoner FM Daros JA Sole RV Elena SF The fittest versus the flattest: Experimental confirmation of the quasispecies effect with subviral pathogens PLoS Pathogens 2006 2 12 1187 1193 10.1371/journal.ppat.0020136 PMC1757203 17196038

[B12] Sanjuan R Cuevas JM Furio V Holmes EC Moya A Selection for robustness in mutagenized RNA viruses Plos Genetics 2007 3 6 939 946 10.1371/journal.pgen.0030093 PMC1892351 17571922

[B13] Gerhart J Kirschner M Cells, embryos, and evolution : toward a cellular and developmental understanding of phenotypic variation and evolutionary adaptability 1997 Boston , Blackwell Science xiii, 642 p.

[B14] Schultes EA Bartel DP One sequence, two ribozymes: Implications for the emergence of new ribozyme folds Science 2000 289 5478 448 452 10.1126/science.289.5478.448 10903205

[B15] Bloom JD Labthavikul ST Otey CR Arnold FH Protein stability promotes evolvability Proceedings of the National Academy of Sciences of the United States of America 2006 103 15 5869 5874 10.1073/pnas.0510098103 16581913 PMC1458665

[B16] Drummond DA Bloom JD Adami C Wilke CO Arnold FH Why highly expressed proteins evolve slowly Proceedings of the National Academy of Sciences of the United States of America 2005 102 40 14338 14343 10.1073/pnas.0504070102 16176987 PMC1242296

[B17] Ancel LW Fontana W Plasticity, evolvability, and modularity in RNA Journal of Experimental Zoology 2000 288 3 242 283 10.1002/1097-010X(20001015)288:3<242::AID-JEZ5>3.0.CO;2-O 11069142

[B18] Sumedha Martin OC Wagner A New structural variation in evolutionary searches of RNA neutral networks Biosystems 2007 90 2 475 485 10.1016/j.biosystems.2006.11.007 17276586

[B19] Turner PE Chao L Sex and the evolution of intrahost competition in RNA virus phi6 Genetics 1998 150 2 523 532 9755186 10.1093/genetics/150.2.523 PMC1460345

[B20] Turner PE Burch CL Hanley KA Chao L Hybrid frequencies confirm limit to coinfection in the RNA bacteriophage phi6 Journal of Virology 1999 73 3 2420 2424 9971826 10.1128/jvi.73.3.2420-2424.1999 PMC104488

[B21] Froissart R Wilke CO Montville R Remold SK Chao L Turner PE Co-infection weakens selection against epistatic mutations in RNA viruses Genetics 2004 168 1 9 19 10.1534/genetics.104.030205 15454523 PMC1448111

[B22] Chao L Fitness of RNA virus decreased by Muller's ratchet Nature 1990 348 6300 454 455 10.1038/348454a0 2247152

[B23] Burch CL Chao L Evolution by small steps and rugged landscapes in the RNA virus phi6 Genetics 1999 151 3 921 927 10049911 10.1093/genetics/151.3.921 PMC1460516

[B24] Burch CL Chao L Epistasis and its relationship to canalization in the RNA virus phi 6 Genetics 2004 167 2 559 567 10.1534/genetics.103.021196 15238511 PMC1470902

[B25] Mindich L Lehman J Cell wall lysin as a component of the bacteriophage phi 6 virion Journal of Virology 1979 30 2 489 496 469991 10.1128/jvi.30.2.489-496.1979 PMC353352

[B26] Gibson G Wagner G Canalization in evolutionary genetics: a stabilizing theory? Bioessays 2000 22 4 372 380 10.1002/(SICI)1521-1878(200004)22:4<372::AID-BIES7>3.0.CO;2-J 10723034

[B27] Hartl DL Taubes CH Compensatory nearly neutral mutations: Selection without adaptation Journal of Theoretical Biology 1996 182 3 303 309 10.1006/jtbi.1996.0168 8944162

[B28] Taddei F Radman M Maynard-Smith J Toupance B Gouyon PH Godelle B Role of mutator alleles in adaptive evolution Nature 1997 387 6634 700 702 10.1038/42696 9192893

[B29] Tenaillon O Toupance B Le Nagard H Taddei F Godelle B Mutators, population size, adaptive landscape and the adaptation of asexual populations of bacteria Genetics 1999 152 2 485 493 10353893 10.1093/genetics/152.2.485 PMC1460623

[B30] de Visser JAGM The fate of microbial mutators Microbiology-Sgm 2002 148 1247 1252 10.1099/00221287-148-5-1247 11988499

[B31] Duffy S Turner PE Burch CL Pleiotropic costs of niche expansion in the RNA bacteriophage phi 6 Genetics 2006 172 2 751 757 10.1534/genetics.105.051136 16299384 PMC1456241

[B32] Duffy S Burch CL Turner PE Evolution of host specificity drives reproductive isolation among RNA viruses Evolution 2007 61 11 2614 2622 10.1111/j.1558-5646.2007.00226.x 17908251 PMC7202233

[B33] Ferris MT Joyce P Burch CL High frequency of mutations that expand the host range of an RNA virus Genetics 2007 176 2 1013 1022 10.1534/genetics.106.064634 17409090 PMC1894571

[B34] Bamford DH Romantschuk M Somerharju PJ Membrane fusion in prokaryotes: bacteriophage phi 6 membrane fuses with the Pseudomonas syringae outer membrane Embo J 1987 6 5 1467 1473 3608985 10.1002/j.1460-2075.1987.tb02388.x PMC553953

[B35] Chao L Rang CU Wong LE Distribution of spontaneous mutants and inferences about the replication mode of the RNA bacteriophage phi 6 Journal of Virology 2002 76 7 3276 3281 10.1128/JVI.76.7.3276-3281.2002 11884552 PMC136006

[B36] Ortlund EA Bridgham JT Redinbo MR Thornton JW Crystal structure of an ancient protein: Evolution by conformational epistasis Science 2007 317 5844 1544 1548 10.1126/science.1142819 17702911 PMC2519897

[B37] Fogarty R Halpin K Hyatt AD Daszak P Mungall BA Henipavirus susceptibility to environmental variables Virus Research 2008 132 1-2 140 144 10.1016/j.virusres.2007.11.010 18166242 PMC3610175

[B38] Abdala N Crowe M Tolstov Y Heimer R Survival of human immunodeficiency virus type 1 after rinsing injection syringes with different cleaning solutions Substance Use and Misuse 2004 39 4 581 600 10.1081/JA-120030059 15115214

[B39] Abdala N Reyes R Carney JM Heimer R Survival of HIV-1 in syringes: effects of temperature during storage Substance Use and Misuse 2000 35 10 1369 1383 10.3109/10826080009148220 10921429

[B40] Dennehy JJ Friedenberg NA Holt RD Turner PE Viral ecology and the maintenance of novel host use American Naturalist 2006 167 3 429 439 10.1086/499381 16673350

[B41] Turner PE Elena SF Cost of host radiation in an RNA virus Genetics 2000 156 4 1465 1470 11102349 10.1093/genetics/156.4.1465 PMC1461356

[B42] Turner PE Chao L Prisoner's dilemma in an RNA virus Nature 1999 398 6726 441 443 10.1038/18913 10201376

[B43] Turner PE Chao L Escape from Prisoner's Dilemma in RNA phage phi 6 American Naturalist 2003 161 3 497 505 10.1086/367880 12699226

[B44] Dennehy JJ Turner PE Reduced fecundity is the cost of cheating in RNA virus phi 6 Proceedings of the Royal Society of London Series B-Biological Sciences 2004 271 1554 2275 2282 10.1098/rspb.2004.2833 PMC1691856 15539353

[B45] Dugatkin LA Cooperation among animals : an evolutionary perspective Oxford series in ecology and evolution 1997 New York , Oxford University Press xvii, 221 p.

[B46] Bruenn JA A structural and primary sequence comparison of the viral RNA-dependent RNA polymerases Nucleic Acids Research 2003 31 7 1821 1829 10.1093/nar/gkg277 12654997 PMC152793

[B47] Bruenn JA Relationships among the positive strand and double-strand RNA viruses as viewed through their RNA-dependent RNA-polymerases Nucleic Acids Research 1991 19 2 217 226 10.1093/nar/19.2.217 2014162 PMC333583

[B48] Mindich L Packaging, replication and recombination of the segmented genomes of bacteriophage phi 6 and its relatives Virus Research 2004 101 1 83 92 10.1016/j.virusres.2003.12.008 15010219

[B49] Sinclair JF Tzagoloff A Levine D Mindich L Proteins of Bacteriophage Phi6 Journal of Virology 1975 16 3 685 695 1159897 10.1128/jvi.16.3.685-695.1975 PMC354716

[B50] Mcgraw T Mindich L Frangione B Nucleotide-sequence of the small double-stranded-RNA segment of bacteriophage-phi-6 - novel mechanism of natural translational control Journal of Virology 1986 58 1 142 151 3754015 10.1128/jvi.58.1.142-151.1986 PMC252886

[B51] Bull JJ Sanjuan R Wilke CO Theory of lethal mutagenesis for viruses Journal of Virology 2007 81 6 2930 2939 10.1128/JVI.01624-06 17202214 PMC1865999

[B52] Anderson JP Daifuku R Loeb LA Viral error catastrophe by mutagenic nucleosides Annual Review of Microbiology 2004 58 183 205 10.1146/annurev.micro.58.030603.123649 15487935

[B53] Rolland M Brander C Nickle DC Herbeck JT Gottlieb GS Campbell MS Maust BS Mullins JI HIV-1 over time: fitness loss or robustness gain? Nature Reviews Microbiology 2007 5 9 2006 2007 10.1038/nrmicro1594-c1 17703224

[B54] Mindich L Calendar R Phages with segmented double-stranded RNA genomes. The Bacteriophages 2006 2nd Oxford ; New York , Oxford University Press xiii, 746 p.

[B55] Mindich L Cohen J Weisburd M Isolation of nonsense suppressor mutants in Pseudomonas Journal of Bacteriology 1976 126 1 177 182 816771 10.1128/jb.126.1.177-182.1976 PMC233272

